# Tsetse fly (*Glossina pallidipes)* midgut responses to *Trypanosoma brucei* challenge

**DOI:** 10.1186/s13071-017-2569-7

**Published:** 2017-12-19

**Authors:** Rosemary Bateta, Jingwen Wang, Yineng Wu, Brian L. Weiss, Wesley C. Warren, Grace A. Murilla, Serap Aksoy, Paul O. Mireji

**Affiliations:** 1 Department of Biochemistry, Biotechnology Research Institute, Kenya Agricultural and Livestock Research Organization, P.O. Box 362, Kikuyu, Kenya; 2 Department of Biochemistry and Molecular Biology, Egerton University, P.O. Box 536, Njoro, Kenya; 3 Department of Epidemiology of Microbial Diseases, Yale School of Public Health, New Haven, CT USA; 4 State Key Laboratory of Genetic Engineering, School of Life Sciences, Fudan University, Shanghai, 200433 China; 5 Ministry of Education Key Laboratory of Contemporary Anthropology, School of Life Sciences, Fudan University, Shanghai, 200433 China; 6 McDonnell Genome Institute, Washington University School of Medicine, 4444 Forest Park Ave., Campus Box 8501, St Louis, MO 63108 USA; 7 Centre for Geographic Medicine Research - Coast, Kenya Medical Research Institute, P. O. Box 428-80108, Kilifi, Kenya

**Keywords:** *Glossina pallidipes*, *Trypanosoma brucei brucei*, Immunity, Challenge

## Abstract

**Background:**

Tsetse flies (*Glossina* spp.) are the prominent vector of African trypanosome parasites (*Trypanosoma* spp.) in sub-Saharan Africa, and *Glossina pallidipes* is the most widely distributed species in Kenya. This species displays strong resistance to infection by parasites, which are typically eliminated in the midgut shortly after acquisition from the mammalian host. Although extensive molecular information on immunity for the related species *Glossina morsitans morsitans* exists, similar information is scarce for *G. pallidipes*.

**Methods:**

To determine temporal transcriptional responses of *G. pallidipes* to *Trypanosoma brucei brucei* challenge, we conducted Illumina based RNA-seq on midgut organ and carcass from teneral females *G. pallidipes* at 24 and 48 h post-challenge (hpc) with *T. b. brucei* relative to their respective controls that received normal blood meals (without the parasite). We used a suite of bioinformatics tools to determine differentially expressed and enriched transcripts between and among tissues, and to identify expanded transcripts in *G. pallidipes* relative to their orthologs *G. m. morsitans.*

**Results:**

Midgut transcripts induced at 24 hpc encoded proteins were associated with lipid remodelling, proteolysis, collagen metabolism, apoptosis, and cell growth. Midgut transcripts induced at 48 hpc encoded proteins linked to embryonic growth and development, serine endopeptidases and proteosomal degradation of the target protein, mRNA translation and neuronal development. Temporal expression of immune responsive transcripts at 48 relative to 24 hpc was pronounced, indicative of a gradual induction of host immune responses the following challenge. We also searched for *G. m. morsitans* orthologous groups that may have experienced expansions in the *G. pallidipes* genome. We identified ten expanded groups in *G. pallidipes* with putative immunity-related functions, which may play a role in the higher refractoriness exhibited by this species.

**Conclusions:**

There appears to be a lack of strong immune responses elicited by gut epithelia of teneral adults. This in combination with a compromised peritrophic matrix at this stage during the initial phase of *T. b. brucei* challenge may facilitate the increased parasite infection establishment noted in teneral flies relative to older adults. Although teneral flies are more susceptible than older adults, the majority of tenerals are still able to eliminate parasite infections. Hence, robust responses elicited at a later time point, such as 72 hpc, may clear parasite infections from the majority of flies. The expanded *G. m. morsitans* orthologous groups in *G. pallidipes* may also be functionally associated with the enhanced refractoriness to trypanosome infections reported in *G. pallidipes* relative to *G. m. morsitans*.

**Electronic supplementary material:**

The online version of this article (10.1186/s13071-017-2569-7) contains supplementary material, which is available to authorized users.

## Background

African Trypanosomiasis constitutes one of the most neglected tropical diseases (NTDs) affecting humans and their livestock with devastating health and economic consequences in Africa [1, 2]. Two forms of the human disease (human African trypanosomiasis, HAT) exist, also known as sleeping sickness. The chronic form in West and Central Africa is caused by *Trypanosoma brucei gambiense*, while the acute form in East and Southern Africa is caused by *Trypanosoma brucei rhodesiense*. The animal disease (animal African trypanosomiasis, AAT), also known as Nagana, is caused by *T. b. brucei*, and related *Trypanosoma vivax* and *Trypanosoma congolense*. All African trypanosomes are transmitted to the mammalian host through the bite of an infected tsetse fly (Diptera: Glossinidae). HAT and AAT remain major public health and veterinary threats, respectively, in most of Africa due to the long adult life of tsetse and exclusive haematophagy of both sexes. Brought under control in the 1960s, HAT re-emerged and resurged to epidemic proportions by the end of the twentieth century due to decreased disease control and surveillance activities. Concerted and collaborative control efforts over the last decade reversed the epidemic trend of the *gambiense* disease, reducing the cases to just 6228 in 2013 [3]. Informed by the progress in HAT control, the WHO Strategic and Technical Advisory Group for NTDs declared a target to eliminate *gambiense* HAT as a public health problem by 2020 and zero incidences of the *rhodesiense* HAT by 2030 [4]. In contrast to *gambiense*, control of *rhodesiense* disease in Central and East Africa is more difficult due to the presence of many animal reservoirs. Although human disease has not been reported in the past decade in Kenya, with the exception of several cases reported in tourists from exposure to infective tsetse bites in game parks [5], there is ongoing risk of re-emergence of *rhodesiense* disease due to the presence of disease in neighboring countries and parasites circulating in wild game and domestic animals, which serve as reservoirs [6, 7]. In contrast, AAT is rampant in livestock inhabiting tsetse-infested areas throughout the continent, including Kenya.

No mammalian vaccines against HAT exist, and there are few available for chemotherapy. Furthermore, treatment is expensive and involves long administration regiments using drugs that have adverse effects [8–10]. Chemotherapy is also problematic due to the widespread and increasing resistance detected in trypanosomes to existing drugs [11], the high cost of treatment and sporadic availability of drugs in areas with the high fly challenge [12]. Tsetse population control efforts, therefore, constitute the cornerstone in disease suppression and eradication efforts. Suppression of tsetse populations relies largely on insecticide-based technologies [13–15]. Also, eradication campaigns integrate a sterile insect technique (SIT) based approach to eliminate residual tsetse populations, as demonstrated in Zanzibar [16]. However, the irradiated male flies released in SIT applications are still capable of transmitting trypanosomes, a challenge that can be surmounted by the development of tsetse release strains refractory to trypanosome infections [17, 18]. The ability to generate parasite-resistant strains requires a better understanding of the molecular interactions that lead to establishment or elimination of parasite infections in tsetse.

The genus *Glossina* consists of three species groups (*Morsitans*, *Palpalis* and *Fusca*), each of which presents differential vector competencies [17]. Flies in the *Palpali*s subgroup are highly refractory to trypanosome infection [19], while those in the *Morsitans* subgroup are more susceptible [20]. Within the *Morsitans* subgroup, there are two closely related species, *G. morsitans morsitans* and *G. pallidipes,* which also show differential susceptibility, the latter being more refractory to infection with trypanosomes [21]. *Glossina pallidipes* is widely distributed in Kenya, and is a vector of AAT, and has transmitted HAT in the past [22, 23].

Trypanosome transmission through the mammalian host and tsetse vector is complex and involves a series of developmental forms. The process of parasite transmission in tsetse begins in the vertebrate host by differentiation of the long slender bloodstream forms (BSF) into non-dividing stumpy forms (ST) [24, 25]. Within hours of ingestion, BSF is readily lysed in the gut while ST parasites differentiate to midgut-adapted procyclic forms (PCFs) [26], which express a non-varying surface coat composed of procyclin proteins [27]. In the majority of flies, trypanosomes are eliminated from the gut within several days post-acquisition, while in a few susceptible individuals PCF parasites survive and establish gut infections [28, 29]. The parasites in these susceptible flies subsequently colonize the proventriculus (cardia) organ and elongate to form mesocyclic trypomastigotes that migrate to the proventriculus where they undergo a complex differentiation as described by Sharma et al. [30] to form short epimastigotes. The short epimastigotes enter the salivary glands where they attach to the epithelial cell and differentiate into metacyclic forms [30]. Mammalian infective metacyclic are transmitted to the next host in saliva as the fly takes a blood meal and differentiate to the BSF that promotes disease [31].

In the laboratory setting, less than 1% of older adults that have received several normal blood meals before receiving infectious parasites become colonized by parasites. This phenomenon may explain why infection prevalence in natural populations is very low even in endemic disease areas [32]. In contrast, newly eclosed adults (termed teneral) are more susceptible to infection when trypanosomes are provided in their first blood meal [33]. The teneral phenomenon has been linked to the immature nature of the peritrophic matrix (PM), which is a chitinous barrier that lines and protects the midgut epithelium from damage by components of the blood meal including the pathogens it may contain [34]. The immature nature of the teneral fly immune system may further contribute to its higher susceptibility. In older adults, the variant surface glycoprotein (VSG) coat proteins of the BSF parasites released into the gut lumen shortly upon acquisition transiently compromises the synthesis of the PM and enables the parasites to bypass the PM barrier [35]. Experimental reduction of PM integrity before parasite acquisition has led to higher infection establishment indicating that PM acts as an initial barrier [36]. Additional factors that influence parasite transmission in adults are midgut proteolytic lectin(s) that may induce transformation of BSF to PCF [37], antimicrobial peptides [38–40], peptidoglycan recognition protein LB [41, 42], TsetseEP protein [43, 44] and reactive oxygen species [45].

Much of the molecular and functional work on tsetse-trypanosome dynamics has been performed with *G. m. morsitans*. However, the molecular dynamics underpinning differential susceptibility in the more refractory vector, *G. pallidipes*, are poorly understood. The differential resistance to infection between these species is more pronounced in the gut than in the salivary glands, such that all *G. pallidipes* with gut infections give rise to mature infections in the salivary glands, while only a proportion of gut infections mature in the case of *G. m. morsitans* [21]. The availability of the annotated whole genome sequences of both *G. pallidipes* [46] and *G. m. morsitans* [47] presents an opportunity to investigate the genetic basis of their differential vector competence. The purpose of this study was to determine the molecular responses of *G. pallidipes* to *T. b. brucei* challenge early in the infection process. Given the strong parasite resistance adult flies express, we analyzed teneral flies (24 h post-eclosion) and evaluated the temporal (24 and 48 h post parasite challenge, hpc) transcriptional responses of the gut tissue and carcass (comprising all other organs) at a time when BSF to PCF differentiation occurs and when PCF parasites are typically eliminated from the gut. This molecular information now forms the foundation on which to build functional investigations to interfere with trypanosome transmission in *G. pallidipes*.

## Methods

### Biological materials

Puparia for *G. pallidipes* were obtained from the Bratislava laboratory in Slovakia and maintained in the insectary at Yale University at 25 °C with 50–60% relative humidity, and adults were fed on bovine blood using artificial membrane feeding method [48]. The *T. b. brucei* strain RUMP 503 used for tsetse challenges was originally isolated from bovines in Nyanza, Kenya (http://tryps.rockefeller.edu/DocumentsGlobal/lineage_EATRO795-LUMP227.pdf ).

### Tsetse fly challenges with trypanosomes

The BSF *T. b. brucei* were expanded in rats, and provided to newly eclosed (one day old) teneral female *G. pallidipes* at a concentration of 2 × 10^6^ cells/ml in bovine blood, while a matching control group received only normal blood. Flies were microscopically dissected in 1× phosphate-buffered saline (137 mM NaCl, 2.7 mM KCl, 10 mM Na_2_HPO_4_, 2 mM KH_2_PO_4_) at 24 or 4 8 h post-challenge (hpc), and ten midguts (minus proventriculi) or carcasses (plus proventriculi) were pooled for each biological sample, respectively. All samples were immediately placed in TRIzol^®^Reagent (Invitrogen, Carlsbad, USA), and subsequently transferred to -80 °C until when required. A total of eight biological samples were collected corresponding to 24 and 48 hpc guts and carcasses from parasite challenged, and normal blood meals, respectively of which four samples were collected from parasite challenged flies and four controls from normal blood meal fed flies.

### Isolation of RNA and RNA-sequencing

Total RNA was isolated using TRIzol reagent following the manufacturer’s protocol, and genomic DNAs (gDNA) digested using Ambion® TURBO DNase™ (Thermo Fischer Scientific, Waltham, MA USA) following manufacturer’s instructions. Removal of the gDNA was confirmed via PCR amplification of the final RNA sample using tsetse specific *beta*-*tubulin* gene primers (Additional file 1: Table S1) as described in Telleria et al. [49] and RNA quality was analyzed by Agilent Bioanalyzer. cDNA was generated by Illumina TruSeq RNA Sample Preparation Kit (Illumina, Hayward, CA, USA) and sequenced on an Illumina HiSeq2000 instrument (paired-end 100 bp) at the McDonnell Genome Institute, Washington University School of Medicine, St Louis, MO, USA. All sequences are available in the Sequence Read Archive (SRA) under study accession numbers SRP090042.

### Identification and validation of differentially expressed (DE) *G. pallidipes* transcripts

Low-quality reads, reads with less than 100 base pairs and adapter sequences were removed by Illumina build software (Illumina, Hayward, CA, USA) in sequence clean up. The resultant raw RNA-Seq reads from each treatment were stored in bam file formats of interleaved FastQ formatted sequences for downstream analysis. Sequence quality in each file was assessed using the FastQC software (http://www.bioinformatics.babraham.ac.uk/projects/fastqc/), and data were filtered for quality using SamToFastq software (http://broadinstitute.github.io/picard/). All filtered reads were aligned to the protein-coding genes of *G. pallidipes* at Vectorbase [46]. The differential expression (DE; differences in expression of transcripts between RNA-Seq libraries) profiles of the transcripts were determined using the RNA-Seq analysis module in the CLC genomic workbench version 8.0 (CLC Bio, Aarhus, Denmark) as described [49]. The profiles were normalized using Kal’s test [50] and compared between challenged and control midguts or carcasses (24 or 48 hpc), respectively. To minimize false positives, transcripts were considered DE between treatments if they had the following criteria: at least a two-fold change, false discovery rate (FDR) corrected *P* < 0.05, at least five reads per kilobase of transcripts per million mapped reads (RPKM), a proxy of gene expression [51] and supported by at least 100 unique read mappings. Most abundant transcripts were considered as those within the 90 percentile in this selection and supported by at least 5000 reads. The fold changes were determined as a ratio of RPKM values between treatments and respective controls and normalized based on the number of reads from each library. Enrichment analysis was conducted to determine enrichment of transcripts within and between two midgut temporal samples and respective carcasses (spatial).

We validated the differentially expressed (DE) profiles of ten randomly selected genes by real-time quantitative PCR (RT-qPCR) analysis from midguts obtained at 48 hpc and controls, respectively. These analyses were conducted using independent biological replicates obtained from dissected midgut and carcass tissues generated under the same experimental conditions as described for the transcriptome samples. Total RNA (1 μg) was reverse transcribed using iScript™ cDNA synthesis kit (BIO-RAD, Hercules, USA), according to manufacturer’s protocol. Transcript expressions were evaluated by RT-qPCR using the gene-specific primers and amplification conditions described in (Additional file 1: Table S1). The expression levels were analyzed with CFX Manager Software version 3.1 (Bio-Rad) and normalized to the *G. pallidipes* housekeeping gene glyceraldehyde 3-phosphate dehydrogenase (*gapdh*) (VectorBase accession number GPAI033271). Fold change in transcript expressions were established by comparing levels of expression in challenged (treatment) relative to unchallenged (control) midguts. Pearson correlation analysis was conducted between fold changes obtained from RT-qPCR to those obtained from the RNA-seq data to estimate our false positive rate.

### Functional annotations of DE transcripts

To identify functions and processes that may be altered by DE putative products, gene ontology (GO), Kyoto Encyclopedia of Genes and Genomes (KEGG) and Wikipathways pathway enrichment analyses were conducted using the web-based gene set analysis toolkit (WebGestalt; Vanderbilt University, TN, USA; http://www.webgestalt.org/ [52]. *Drosophila melanogaster* genes were used as a proxy for *G. pallidipes* where *D. melanogaster* homologs of the *G. pallidipes* differentially induced or suppressed genes were employed. Hypergeometric test, Benjamini & Hochberg multiple test adjustment [53] and *P* < 0.05 cut-off values were employed to separate and identify significant functions and pathways. Additional functional annotations of DE gene sets were performed using BLASTx [54] to compare nucleotide sequence to the non-redundant protein database at National Centre for Biotechnology Information (NCBI), GO and Interpro databases using Blast2GO™ software [55, 56]. An e-value of 0.001 was used to perform the BLAST and annotation steps while mapping was carried out by default settings. *Drosophila melanogaster* transcripts encoding putative immune-specific and associated proteins were acquired from FlyBase [57] as previously described [47, 49] and were used to identify their potential homologs among the DE transcripts by tBLASTx [54] homology searches. Heatmaps of gut DE transcripts at 24 and 48 hpc were developed by comparing fold changes of respective RPKM values using Complex Heatmaps Bioconductor R package [58] by employing “maximum” and “ward.D” methods within the package. Orthology groups containing *G. pallidipes* specific gene expansions, as determined by the Ensembl compara pipeline [59], were retrieved from Vectorbase [46]. These genes were functionally annotated as previously described using BLASTx and Blast2GO™ software. The DE profile of the orthologs was analyzed between the 24 and 48 hpc datasets using Complex Heatmaps Bioconductor R package [58] where only orthologs supported by at least 100 reads and more than 1 RPKM were considered.

## Results

### Global expression profiling of *G. pallidipes* responses following challenge with *T. b. brucei*

**Fig. 1:**
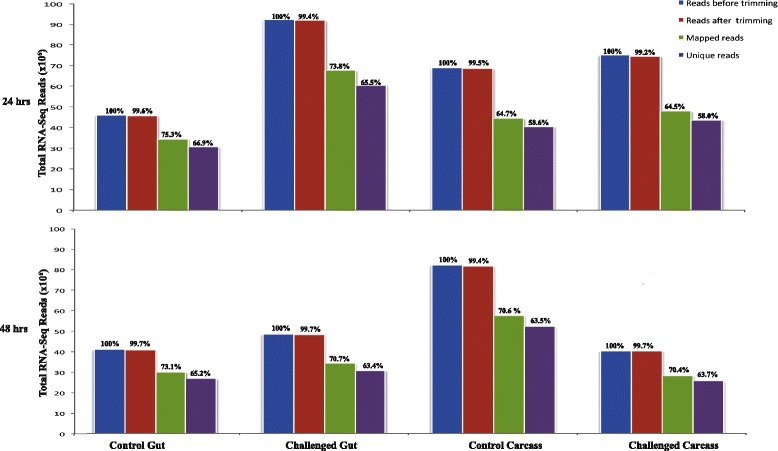
Summary of processing and mapping statistics of RNA-Seq reads from teneral female *G. pallidipes* gut and carcass 24 or 48 h post-challenge with *T. b. brucei*

**Fig. 2:**
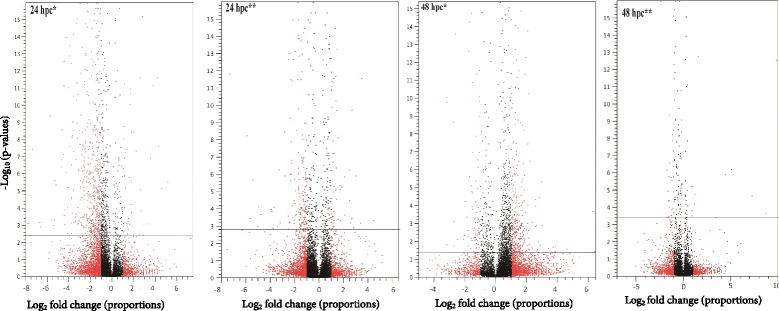
Volcano plots of expressed transcripts in *G. pallidipes* 24 or 48 h post-challenges with *T. b. brucei.* Red spots indicate differentially expressed transcripts with two (Log2 = 1) or more fold-change (x-axis) and high statistical significance (-log10 of *P*-value, y-axis). Horizontal blue line shows where Bonferoni FDR corrected *P*-value = 0.05 with spots above the line having < 0.05 and spots below the line having > 0.05. Spots having a fold-change less than 2 are shown in black. Only the genes denoted with red spots above the blue line are considered significant. *Abbreviations*: hpc, hours post-challenge; hpc*, challenged *vs* unchallenged gut; hpc**, challenged *vs* unchallenged carcass

Processing of the RNA-Seq data yielded 43 to 92 million reads in the 24 and 48 hpc midgut and carcass libraries (Fig. 1), of which 64.5–75.3% could be mapped to *G. pallidipes* genes, respectively (Fig. 1). At least 89% of the mapped reads were unique to specific genes. To validate the transcriptome data, the expression profiles of ten randomly selected transcripts were obtained using RT-qPCR from RNA extracted from independent biological samples of *G. pallidipes* parasite challenged and control guts, respectively. The comparison revealed a Pearson correlation coefficient (*R* = 0.766) and goodness of fit (R^2^ = 0.586) (Additional file 2: Table S2, Figure S1) for the ten genes evaluated, indicative of a valid transcriptome [49]. When challenged guts and carcasses were compared to controls, more DE transcripts were found at 24 hpc than 48 hpc (Fig. 2). When fold changes and significance (*P*-value) based global dispersion patterns of DE transcripts expressed in challenged and control guts and carcasses were analyzed, more transcripts were suppressed than induced at 24 hpc relative to 48 hpc (Fig. 2).

### Gut-enriched transcripts and putative protein-protein interactions in *G. pallidipes* challenged with *T. b. brucei*

**Fig. 3:**
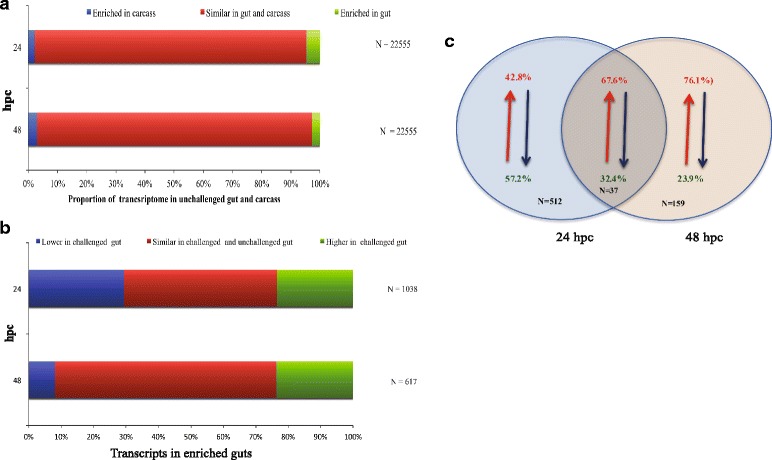
Summary of temporal differential enrichments of transcripts detected in *G. pallidipes* 24 or 48 h post-challenge with *T. b. brucei*. **a** Gut and carcass tissue specific distribution of transcripts detected*.* **b** Expression profiles of gut enriched trancripts in *G. pallidipes* challenged *vs* unchalleged with *T. b. brucei*. **c** Temporal expression profile of gut specific *G. pallidipes* genes differentiallly expressed. *Abbreviation*: hpc, hours post-challenge

Under normal and parasite challenging conditions (Fig. 3), most of the transcripts (> 93%) were expressed at similar levels in control versus treatment datasets, despite the temporal change. Only 4.6% (1038) and 2.7% (617) of transcripts were induced in the gut 24 and 48 hpc, respectively (Fig. 3a). The expression profiles of the gut-enriched transcripts were analyzed to understand potential physiological changes that may influence parasite infection processes. The gut is the initial immune-associated contact tissue by the parasite, with the carcass providing immunological responses later in the infection [39]. Temporal analysis of gut induced transcripts showed that more transcripts were suppressed at 24 hpc than at 48 hpc in the presence of the parasite (Fig. 3b, c). Only 37 DE transcripts were shared between the 24 and 48 hpc datasets and expression most of these transcripts (25) was up-regulated (Fig. 3c).

A web-based gene set analysis toolkit [51] was used to reveal functions and pathways induced or suppressed at 24 or 48 hpc (Additional file 3: Table S3). The induced transcripts in the 24 hpc gut dataset were predominantly associated with carboxylic acid metabolic processes and negative regulation of RNA post-translational modification, while those at 48 hpc were associated with axonogenesis, cytoskeleton organization and response to a stimulus. The 24 hpc suppressed gut-associated transcripts were dominated by chitin metabolism, response to oxidative stress and associated genes, while the analysis did not associate any specific pathway with 48 hpc suppressed transcripts. In the carcass, transcripts associated with chitin metabolism were induced at 24 hpc and suppressed at 48 hpc (Additional file 4: Table S4). The dissected gut tissues we studied did not contain the cardia organ; hence the cardia specific transcriptional responses that are involved in PM synthesis are represented in the carcass dataset, while our method allowed for analysis of midgut-specific responses. Pathways associated with induction of amino acid (arginine, proline tryptophan), purine and glycogen/glucose metabolism, as well as folate and terpenoid backbone biosynthesis, were among those enriched in the 24 hpc gut dataset.

### Expression of immunity associated genes in *G. pallidipes* gut 24 and 48 hpc

When DE transcripts were interrogated through BLAST analysis, 139 transcripts were associated with an immune function in either *G. m. morsitans* and/or *D. melanogaster*. Of these, 27.3% were induced, and 45.3% were suppressed at 24 hpc, while 21.6% were induced and 5.8% were suppressed at 48 hpc (Additional file 5: Table S5). Transcripts predominating the expression profile included induction of *CD109 antigen*, and suppression of both *trypsin epsilon* and *serine protease sp24d* (Additional file 5: Table S5). Expression of Tld domain-containing protein 2, ejaculatory bulb-specific protein 3 and endocuticle structural protein/glycoprotein encoding genes were also suppressed at 24 hpc. Similarly, expression of chymotrypsin-1, AP2-associated protein kinase 1, myosin heavy non-muscle, transferrin and croquemort coding genes were induced, while those of lectin subunit alpha was suppressed at 48 hpc. Transcripts for the serpin 3 and toll proteins associated with the toll signalling pathway were induced and suppressed, respectively at 24 hpc. Similarly, expression of the immune deficiency (Imd) pathway associated genes mask and peptidoglycan recognition protein LC (PGRP-LC) were induced and suppressed, respectively, 24 hpc, while notch was the only induced transcript in the Imd pathway at 48 hpc. PM-related putative products reduced in the gut included chondroitin proteoglycan A 1 and cysteine transfer RNA ligase gene.

### Heatmap of DE gut transcripts at 24 and 48 hpc

**Fig. 4:**
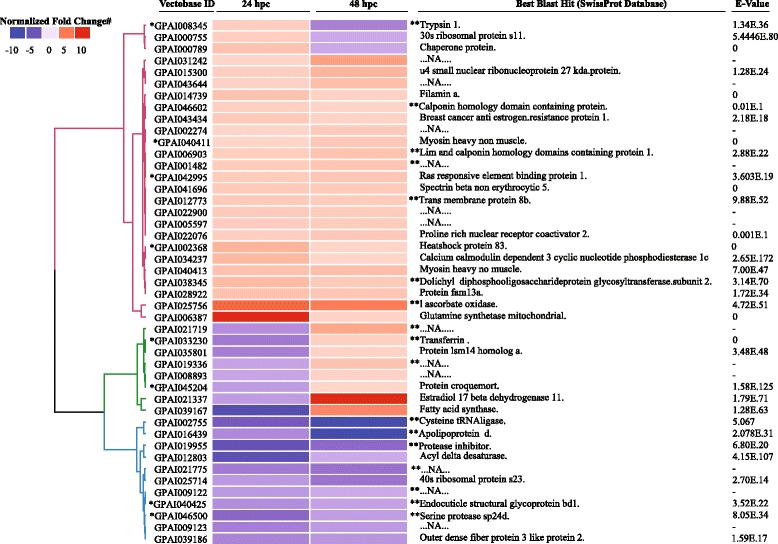
Heatmap of differentially expressed transcripts in *G. pallidipes* guts challenged with *T. b. brucei* for 24 or 48 h in relation to their respective controls. *Key*: *, immunity associated transcript; **, secreted protein; #, relative to respective controls

The heatmap of DE transcripts at 24 and 48 hpc gut datasets revealed distinct temporal gene expression profiles (Fig. 4). In particular, trypsin-1, 30S ribosomal proteins II, chaperone protein, heat shock protein 83 and glutamine synthetase coding transcript abundances were higher at 24 hpc relative to 48 hpc. Transcripts induced at 48 hpc relative to 24 hpc included estradiol 17-beta-dehydrogenase 11, fatty acid synthase, protein croquemort and several hypothetical proteins. Among the temporal DE transcripts, only trypsin-1 and protein croquemort were immune-associated (Fig. 4).

### Abundant transcripts 24 or 48 hpc

Analysis of the most DE (> 90 percentile) and enriched (supported by at least 5000 reads) transcripts 24 hpc relative to the unchallenged control midgut libraries revealed induction of functions associated with lipid remodeling/lipogenesis, proteolysis, the urea cycle, carnitine trafficking, collagen metabolism, apoptosis, and cell growth/differentiation (Additional file 6: Table S6). The majority of these transcripts did not encode secreted proteins (77%) and/or products associated with immunity (73%). Functions associated with transcripts that were suppressed included nervous system development, neurotransmitter transport/cellular calcium ion hemostasis and cuticular structure.

At 48 hpc, most induced transcripts were associated with pathways involved in embryonic growth and development, muscle/motility function, tumour suppression, proteosomal degradation of target proteins (serine endopeptidases and related enzymes), mRNA translation and neuronal development. Similar to 24 hpc, only 33% of these transcripts encoded secreted products, and 10% were associated with immunity. Of note, pathways associated with ATP-dependent degradation of ubiquitinated proteins and cell proliferation/migration were suppressed, and only 50% of these transcripts encoded secreted or immune associated products.

### Annotations of *G. m. morsitans* orthologs expanded in *G. pallidipes*

**Fig. 5:**
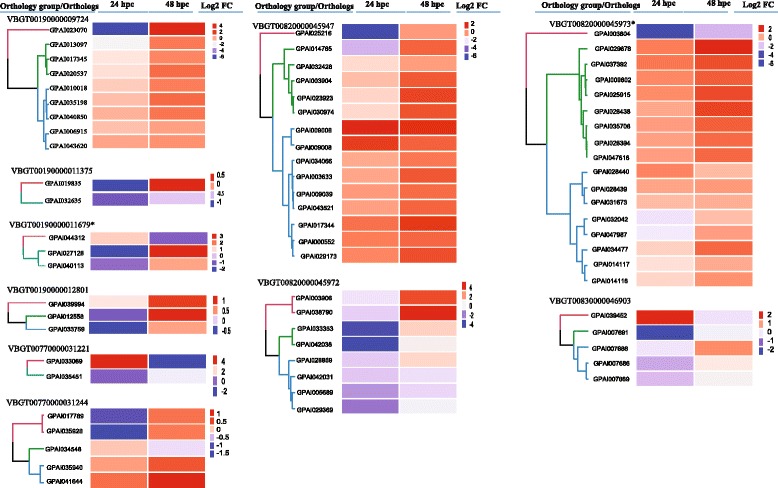
Expression profiles of expanded *G. m. morsitans* orthologs in *G. pallidipes* midguts 24 or 48 h post-challenge with *T. b. brucei* in relation to their respective controls. Expression cut-off: reads per kilobase per million (RPKM) > 1 and unique read mapping > 100. *Abbreviations*: hpc, hours post-challenge; FC, fold change; *, with Immune associated GO terms

Analysis of *G. m. morsitans* orthologous groups in Vectorbase [46] revealed a 51–83% expansion (proportion of *G. pallidipes* orthologs for each respective *G. morsitans* ortholog) of ten gene families in *G. pallidipes* relative to *G. m. morsitans* (Additional file 7: Table S7, Fig. 5). Predicted functions of these gene products were determined by tBLASTx [54] homology searches against the NCBI nr protein database. This analysis associated the orthologous groups containing expanded gene members with DNA replication licensing factors, N-acetyl-D-glucosamine kinase, brunelleschi, transportin-3, importin-13, serine-threonine- kinase, mastermind 3, DEP domain-containing protein, MICOS complex subunit, kinesin, fatty acyl- reductase, several transcriptional regulators and the zinc finger protein weekly. No homolog was identified for one of the orthologous groups (VBGT00190000011679), which together with the transcriptional regulators (VBGT00820000045973) were associated with immunity by their respective GO terms biological processes annotations. Analysis of the expression profiles of expanded orthologous groups in the *G. pallidipes* midgut showed general inductions of the orthologs by trypanosome challenge at both time points post-challenge (Fig. 5).

## Discussion

This study reports on the molecular responses of *G. pallidipes* to *T. b. brucei* at a critical moment in the infection process when most trypanosomes are typically cleared, with a few surviving to establish permanent infections in the fly midgut. *G. pallidipes* is more refractory to trypanosome establishment than *G. m. morsitans*, and therefore we used a transcriptomic approach to study the early molecular responses in *G. pallidipes* upon challenge with *T. b. brucei*. We analyzed host responses from two different fly compartments: the gut where epithelial immune responses can limit parasite survival, and the carcass that is involved in systemic immunity and metabolic responses. We also used these datasets to identify host responses that are preferentially enriched in the gut. We established that gut transcripts associated with metabolic processes dominated the early (24 hpc) responses; with immune-associated gene expression beginning to be detected in the later (48 hpc) responses. These findings suggest that in the fly’s teneral state, parasites encounter minimal immunological challenge upon entering the gut, which potentially permits the differentiation and survival of the parasite from BSF to PCF forms early in the infection. Our observations may reflect the immature nature of the gut immune responses in teneral tsetse. However, at 24 hpc, our findings of induced transcripts, such as serine protease sp24d, which is associated with immunity, may also suggest a process of systematic suppression of host immunity. Such a phenomenon, during which parasites inhibit tsetse immunity to facilitate their colonization of the fly, is evident in the case of tsetse cardia responses, and reduced PM formation, following parasite acquisition [35]. Similarly, induction of transcripts associated with cytoskeletal reorganization may indicate modifications of tsetse gut physiology that can influence parasite differentiation processes or be a response to parasite-induced damage of host epithelia [60, 61]. We also noted suppression of oxidation-reduction processes at 24 hpc, as shown by reduction of transcripts associated with calcium binding proteins. In another study in teneral *G. p. gambiensis* challenged with *T. b. gambiense,* proteome analysis revealed induction of these processes at a later time in the infection process, 72 hpc [62]. Suppression of oxidation-reduction processes is thought to reflect oxidative stress resulting from a large amount of heme present in the blood meal and/or a response to the invading parasites [44]. Oxidative stress responses are reported in a variety of insects upon pathogen challenge, including tsetse [35, 39, 63, 64]. Suppression of these responses upon parasite entry into tsetse’s gut suggests promotion of parasite survival or differentiation early in the infection process. Gut responses from older adult *G. m. morsitans* at 48 and 72 hpc also noted induction of ROS responses as part of the immune arsenal that may result in parasite refractoriness [35].

In teneral flies, we also noted increasing immune responses to parasite challenge at 48 hpc, suggesting gradual maturation of the immune system. The observed induction of transcripts related to the keratinocyte signalling pathway, associated with apoptosis [65], signals recognition of dead cells or parasite antigens by the host defences during parasite challenge. Parasite products are known to compromise the integrity of host gut physiology during the early course of infection in older adult flies. Specifically, mammalian parasite surface coat VSG proteins, which are released into the lumen, modify host transcriptional responses transiently, thus reducing PM barrier integrity in adult flies [35]. The integrity of the PM is an important barrier that limits parasite infections in older adults [36]. In our analysis in teneral flies, we also noted a reduction of PM-related products in the gut, which includes chondroitin proteoglycan A 1 involved in chitin binding and the cysteine transfer RNA ligase gene. Besides the PM-associated products, we also noted alterations in transcripts associated with chitin metabolism, the main structure of the PM backbone. While chitin metabolism was induced in the carcass at 24 hpc, we noted suppression of the same transcripts at 48 hpc in the gut. The transcript induction observed at 24 hpc in the carcass could be linked to insect growth and morphogenesis [66] as we used teneral tsetse with an underdeveloped exoskeleton. However, suppression of these transcripts at 48 hpc may be tied to suppression of chitin metabolism in the gut associated with impaired PM structure to facilitate trypanosome escape to the ectoperitrophic space. Lack of mature immune responses in the teneral state can further facilitate the establishment of these infections in the teneral state, while they would be more effectively cleared from the more immuno-competent gut of mature adults.

The greater parasite refractoriness reported in *G. pallidipes* in relation to *G. m. morsitans* [21] may be influenced by the ten orthologous groups (gene families) that are expanded in the *G. pallidipes* genome relative to *G. m. morsitans*. Most of these gene expansions are associated with immune pathways where they appear to enhance innate immunity in the host. The mastermind 3 gene orthologs are characterized components of the immune-associated notch signalling pathway [67]. The DEP domain-containing protein orthologs are involved in intra cellular signal transduction in regulation of immune responses by the mTOR signalling pathway [68]. The zinc finger weckle protein is a component of the toll signalling pathway [69]. Additionally, two expanded orthologous groups (VBGT00190000011679 and VBGT00820000045973) are associated with immune function by their respective GO terms and appear to regulate the immune responses at the nuclear level, an observation underscored by other nucleus based cell signalling components that are encoded by the rest of ortholog gene expansions. These activities include enhanced kinesin associated intracellular transport [70] that is associated with activation of immune cells in idiopathic inflammatory myopathies [71], duplication of genomic DNA by DNA replication licensing factors [72], regulatory roles of N-acetyl-D-glucosamine kinase in gene expression [73], meiosis cytokinesis by brunelleschi [74], and nuclear import of splicing factors through nuclear pore complexes by transportin [75] and importin-13 [76]. In our transcriptomes, these genes are upregulated in a midgut preferential manner. The enhanced temporal expression of these orthologous families upon trypanosome challenge underscores their putative immune responsive role in *G. pallidipes*, which can be confirmed through functional genomic studies in the future.

Follow up studies with a spectrum of pathogen and trypanosome developmental forms (BSF/ PCF) may provide insights on whether these response patterns are pathogen-specific and the potential role that the BSF - PCF transformation has on these patterns. Additionally, the role of the tsetse microbiome in modulating these responses also merits investigation, as factors influencing parasite establishment in the fly midgut, are not only genetic. Both *Wigglesworthia glossinidia* and *Sodalis glossinidius*, which are prominent members of tsetse’s indigenous microbiota, exert a strong influence on tsetse’s vector competency [77–79].

## Conclusions

In conclusion, there appears to be a lack of strong immune responses elicited by gut epithelia of teneral adults. This in combination with a compromised PM at this stage during the initial phase of *T. b. brucei* challenge may facilitate the increased parasite infection establishment noted in teneral flies relative to older adults. Although teneral flies are more susceptible than older adults, the majority of tenerals are still able to eliminate parasites infections. Hence, more robust responses elicited at a later time point, such as 72 hpc may clear parasite infections from the majority of flies. The expanded *G. m. morsitans* orthologous groups in *G. pallidipes* may also be functionally associated with the enhanced refractoriness to trypanosome infections reported in *G. pallidipes* relative to *G. m. morsitans*.

## Additional files

Additional file 1: Table S1. Primers utilized on *G. pallidipes* DNA and cDNA PCR. (DOCX 14 kb)

Additional file 2: Table S2. Validation of *G. pallidipes* RNA Seq data with qPCR RNA-seq expression values (log2 ratios) for ten genes plotted against qPCR values (log2 ratios). **Figure S1.** RNA-seq expression values (log_2_ ratios) for ten genes plotted against qPCR values (log_2_ ratios). (PDF 1742 kb)

Additional file 3: Table S3. Canonical gene set enrichment analysis (GSEA) of significantly differentially expressed transcripts in the female *G. pallidipes* 24 or 48 h post-challenge with *T. B. brucei*. (XLSX 14 kb)

Additional file 4: Table S4. Major (top ten) secreted/non-secreted differentially expressed genes in carcases of teneral *G. pallidipes* tsetse flies 24 or 48 h post-challenge by *T. b. brucei* parasites. * = treatment transcriptome; ** = reference trancriptome. (XLSX 351 kb)

Additional file 5: Table S5. Putative immune responsive genes in *G. pallidipes* guts challenged with *T. b. brucei*. (XLSX 43 kb)

Additional file 6: Table S6. Predominantly (90 percentile) differentially expressed genes in guts and carcasses of teneral *G. pallidipes* tsetse flies challenged with *T. b. brucei* parasites 24 or 48 h. * = treatment transcriptome; ** = reference transcriptome. (XLSX 25 kb)

Additional file 7: Table S7. Orthology groups expanded in *G. pallidipes* in relation to *G. m. morsitans* and their associated Blast annotations and gene ontology (GO) terms. (XLSX 18 kb)

## Abbreviations

AAT: Animal African trypanosomiasis
BLAST: Basic Local Alignment Search Tool
BST: Bloodstream forms
cDNA: Complementary deoxyribonucleic acid
DE: Differential expression
DNA: Deoxyribonucleic acid
FDR: False discovery rate
gDNA: Genomic deoxyribonucleic acid
GO: Gene ontology
HAT: Human African Trypanosomiasis
hpc: Post-challenge
IMD: Immune deficiency
KEGG: Kyoto encyclopedia of genes and genomes
mRNA: Messenger ribonucleic acid
NCBI: Centre for Biotechnology Information
NIH: National Institutes of Health
NTD: Neglected tropical diseases
PCF: Procyclic forms
PCR: Polymerase chain reaction
PGRP-LC: Peptidoglycan recognition protein LC
PM: Peritrophic matrix
RNA: Ribonucleic acid
RPKM: Reads Per Kilobase of transcripts per Million mapped reads
RT-qPCR: Real-time quantitative PCR
SF: Stumpy forms
SIT: Sterile insect technique
VSG: Variant surface glycoprotein
WHO: World Health Organization
