# Supplementary material for: Tsetse fly (Glossina pallidipes) midgut responses to Trypanosoma brucei challenge
Source: Parasit Vectors. 2017 Dec 19;10:614. doi: 10.1186/s13071-017-2569-7 (PMC5738168; doi:10.1186/s13071-017-2569-7)
Supplement: Supplementary file 1 — Primers utilized on G. pallidipes DNA and cDNA PCR. (DOCX 14 kb) [file 13071_2017_2569_MOESM1_ESM.docx]

**Additional file 1: Table S1.** Primers utilized on *G. pallidipes* DNA and cDNA PCR

| **Putative tsetse Gene ID** | **Vectorbase Accession ID** | **Forward primer** | **Reverse primer** |
| --- | --- | --- | --- |
| Tsetse β-tubulin | GPAI022614 | ACGTATTCATTTCCCTTTGG | AATGGCTGTGGTGTTGGACAAC |
| GAPDH | GPAI033271 | CTGATTTCGTTGGTGATACT | CCAAATTCGTTGTCGTACCA |
| Glutamine Synthase | GPAI006387 | TGGTGGAAGCTCATGCCTTG | CAACGCTGGGTCCTACTTGG |
| Heat Shock protein 83 | GPAI002368 | GGAATGACCAAGCCGGATTTG | CAGCAATCAAATACGCGGAATAG |
| Multi Copper oxidase | GPAI025756 | ACTGAGCCAATGCCACCTATACTG | GCACACTTACCGCAAGCAACTC |
| Pyruvate Carboxylase | GPAI003647 | GAGGGTATGGGCATACGTTTG | TTGTAAGTCGCTGGCATGTGAG |
| Chitinase 4 | GPAI022616 | GCTATGTATGGGCACTCTTTTCAG | TGTCTCGCAGATTTCATTGTAACC |
| Transferrin 1 | GPA1033230 | CATCCACGCTAACAACTA | AAGTAGGTTCCACAACAG |
| Tep2 | GPAI040205 | CCAACGCCCATTACCCCTACA | GCCCCGCTGAAGGTGGTA |
| Serpin 4 | GPAI011576 | CGGCTATGGCTCGCTTAG | TGTCGCTTTTCTCGTATTGC |
| Serpin 6 | GPAI011576 | AATGAGGCTGCTGCTGCTAG | CGCCCAGTTCCCTTTGAAATG |
| PGRP-LB | GPAI047520 | CAACAACAACCCAAAAGG | GAGTTGGTACTGCCGATGT |
