# Supplementary material for: Tsetse fly (Glossina pallidipes) midgut responses to Trypanosoma brucei challenge
Source: Parasit Vectors. 2017 Dec 19;10:614. doi: 10.1186/s13071-017-2569-7 (PMC5738168; doi:10.1186/s13071-017-2569-7)

Additional file 2: Table S2. Validation of G. pallidipes RNA Seq data with qPCR RNA-seq expression values (log2 ratios) for ten genes plotted against qPCR values (log2 ratios)

Gene	Accession Number	qPCR Fold Change	Log2 of qPCR	RNA-seq Fold Change	Log2 of RNA-seq
Tep2	GPAI040205	3.61	1.85	2.76	1.46
Multicopper oxidase	GPAI025756	5.98	2.58	4.2	2.07
Glutamine synthase	GPAI006387	4.18	2.06	2.08	1.06
Heat shock protein 83	GPAI002368	4.77	2.25	2.05	1.04
Pyruvate carboxylase	GPAI003647	1.84	0.88	2.11	1.08
Transferrin	GPA1033230	2.34	1.23	2.19	1.13
Serpin 4	GPAI011576	3.20	1.68	2.44	1.28
Serpin 6	GPAI011576	3.20	1.68	1.16	0.21
PGRP-LB	GPAI047520	1.47	0.56	1.32	0.40
Chitinase	GPAI022616	9.19	3.20	6.76	2.76

Additional file 2: Figure S1. RNA-seq expression values (log_2 ratios) for ten genes plotted against qPCR values (log_2 ratios)

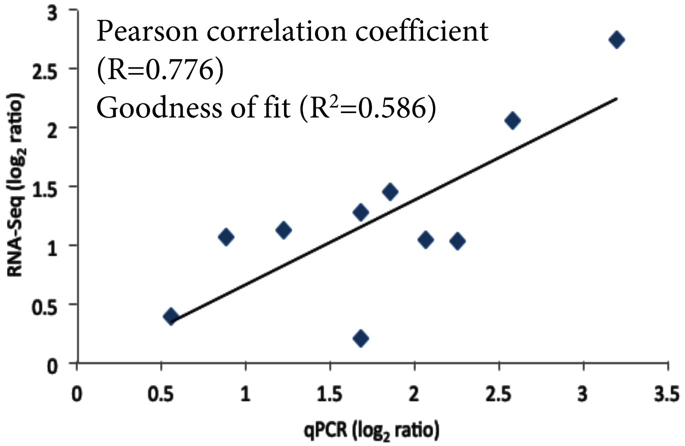

Supplement: Supplementary file 2 — Validation of G. pallidipes RNA Seq data with qPCR RNA-seq expression values (log2 ratios) for ten genes plotted against qPCR values (log2 ratios). Figure S1. RNA-seq expression values (log2 ratios) for ten genes plotted against qPCR values (log2 ratios). (PDF 1742 kb) [file 13071_2017_2569_MOESM2_ESM.pdf]
